# Characterization of Hybrid Composites with Polyester Waste Fibers, Olive Root Fibers and Coir Pith Micro-Particles Using Mixture Design Analysis for Structural Applications

**DOI:** 10.3390/polym13142291

**Published:** 2021-07-13

**Authors:** Muhammad Rizwan Tufail, Hafsa Jamshaid, Rajesh Mishra, Uzair Hussain, Martin Tichy, Miroslav Muller

**Affiliations:** 1 Faculty of Textile Engineering, National Textile University, Faisalabad 37610, Pakistan; formanite819@gmail.com (M.R.T.); hafsa@ntu.edu.pk (H.J.); uzair@ntu.edu.pk (U.H.); 2 Department of Material Science and Manufacturing Technology, Faculty of Engineering, Czech University of Life Sciences Prague, Kamycka 129, 165 00 Prague 6-Suchdol, Czech Republic; martintichy@tf.czu.cz (M.T.); muller@tf.czu.cz (M.M.)

**Keywords:** hybrid composite, polyester waste fiber, olive root fiber, coir pith filler, building materials, mixture design analysis

## Abstract

In the present work, hybrid composites were developed by using polyester waste fibers along with natural origin materials: olive root fibers and coir pitch filler. Such composite panels can be used as a potential alternative for fiber glass sunshade panels and room dividers in buildings. Hybrid composites were fabricated by mixing polyester waste fibers and olive root fibers in different ratios (0:100, 33:67, 67:33 and 100:0). Coir pith micro-particles with an average size of 312 d.nm were used as filler in the polyester matrix at three different levels (0%, 5%, and 10%) of the overall matrix weight. Mechanical properties, e.g., tensile strength, flexural strength and impact strength, thermal properties, e.g., coefficient of linear thermal expansion, thermo-gravimetric analysis (TGA) and environmental properties, e.g., water absorption, loss of density after exposure to weathering were characterized. For comparison purposes, a commercially available fiber glass sunshades sample was also investigated. Mixture design analysis was used to optimize the ratio of all components in the composite. Graphical comparison of experimental results using regression models showed a high degree of correlation. An optimized formulation of composite with an objective of maximization of tensile strength, flexural strength, impact strength and minimization of water absorption, density loss, as well as coefficient of linear thermal expansion, was determined at 70.83 wt%, 15.15 wt%, and 14.01 wt% of polyester waste fibers, olive root fibers and coir pith micro-fillers, respectively. Overall, it can be concluded that the developed hybrid composites from waste fibrous materials can be used as a promising alternative and a value-added application in buildings and construction purposes.

## 1. Introduction

Composite material performance and properties depend on the properties of individual components, mixture proportions and their inter-facial compatibility. The composite materials primarily consist of two constituents, one of which is the reinforcement material that could be treated with chemicals for surface modification [1] in order to improve binding and handling properties. The second component is the matrix which serves to protect the reinforcement material from environmental and external damage by transfer of the load [2]. In addition, they may contain a third component called “fillers” which are mixed with polymeric matrix in order to improve its mechanical and thermal properties. Fillers are used for reducing the overall weight and cost of the composite while enhancing its performance [3,4]. Natural fiber-based composites are environmentally superior in specific fields of application [5]. The global interest in natural fiber-based composites is growing because they are alternatives for synthetic or man-made fibers such as glass or carbon [6]. Natural fiber reinforcements are a renewable resource, and their production requires little energy. They have very low carbon dioxide emissions and are not dependent on petroleum-based precursors. Sisal, bamboo, jute, and coir are widely used as engineering materials in different industries [7,8].

A proper material design helps in achieving a balance in performance and cost for application in numerous fields ranging from the building industry to the automobile industry [9,10]. Recently, different industries are looking into the replacement of polymer or glass fiber composites. The natural fiber-based composites have relatively lower mechanical performance and higher water absorption properties compared to the glass or carbon fiber-based composites. Therefore, a hybrid fabrication method is used to produce composite products with improved mechanical, thermal, water resistance, and water absorption properties. In this technique, composite materials are manufactured by using two or more reinforcing materials in a single matrix or two polymers blended matrices with single natural fiber reinforcement. The reinforcing materials can be made of natural or synthetic fibers depending on the required performance level [11,12]. Polyester fibers are consumed in different textile applications. Their origin is from petroleum-based materials which are not sustainable, and their biodegradability is another issue. Polyester waste fibers can be recycled in order to reduce environmental pollution by not putting them into landfills. That would prevent the chemicals from leaching into the soil. Reusing is another option that is more energy-efficient than recycling, especially when it can be used for value-added products. Franciszczak et al. revealed that the injection-molded composite of short PET fibers reinforced to PETG (Polyethylene terephthalate glycol) matrix display good impact strength on the same level of polypropylene reinforced by glass fibers [13]. Wu et al. determined that the self-reinforced PET composite of high tenacity PET as reinforcement and copolymerized PET as matrix show excellent flexural and impact behavior [14]. Ahmad et al. studied the composite of hybrid fabric by the interlacement of warp (hemp yarn) and weft (PET yarn) produced by vacuum infusion process with epoxy resin. Such composites display an enhancement in tensile strength [15]. Composite samples were produced from six layers of woven kenaf fabrics arranged at symmetric angles (0°, 45°, 90°) and impregnated with PET in POM (Polyoxymethylene) matrix. The hybrid composite samples showed increases in the modulus and strength [16]. Wu et al. studied that self-reinforced PET composite of recycled PET homopolymer filaments serving as the reinforcements and copolymerized PET (mPET) filaments as the matrix exhibit improved resistance to creep deformation [17]. Composites prepared by hand layup and compression molding of coir pith/nylon/epoxy showed that chemical treatment improves the water resistance and offers optimum retention of impact strength in composites [18]. Essabir et al. developed polypropylene hybrid composites by using coir fibers and coir shell micro-particles under injection molding. The findings indicate that coir shell particles have low thermal degradation compared to coir fiber [19]. Islam et al. concluded that hand layup of coir mat/polyester composite with coir powder as filler with varying content of (10, 20, 30, 40, 50, and 100%) shows an improvement in mechanical properties up to 30% and further addition of filler results in deterioration of mechanical properties [20]. Hybrid composite with coir pith/nylon fabric/epoxy made by hand lay-up showed that NaOH treatment of coir pith increases the composite’s impact strength and water resistance [21,22]. A hybrid composite of coir pith, rice husk, and groundnut shell was prepared with epoxy resin. The results revealed that the hybridization of micro-particles greatly influenced the tensile and flexural properties of the composites [23,24]. Composites based on recycled PET fibers with an average length of (2 to 20 mm) in the polyester matrix exhibited good dispersion, interfacial adhesion, and high affinity of PET fibers with matrix leading to improved mechanical performance [25]. Abdulla et al. developed the hybrid composite of kenaf and PET fibers reinforced POM using compression molding technique and exposed the samples to ultraviolet penetration, moisture, and water spray in the weathering chamber. The increasing PET contents indicated better retention in mechanical properties when exposed to UV and moisture as compared to natural fiber-rich composites. Thus, hybrid composites are more suitable for outdoor applications [26]. The hybrid composite of kenaf/PET fiber in the POM (polyoxymethylene) matrix was investigated to study the mechanical as well as moisture absorption properties. The impact strength of composites using longer fibers was found to be higher than composites reinforced with short fibers [27]. Studies on composites reinforced with glass fibers, waste polyethylene terephthalate fibers and kenaf fibers developed by compression molding concluded that the tensile strength of glass fiber-reinforced PET waste and kenaf hybrid composites was higher than the pure PET waste composites. They can be used for construction materials like wall and partition materials [28]. The injection-molded polypropylene composites were prepared with distinct mixtures of the wood flour and the olive mill sludge (OMS). With increasing olive mill sludge, the water-resistance of composites increases. However, the flexural characteristics deteriorate with the increase in OMS flour content [29].

Mixture design analysis is used where the response depends on mixture proportions of various components and not on the absolute amount of incorporating materials. For example in an alloy, the mechanical properties may depend on variable proportions of the various metals but not on the absolute amount of each component. The mixture design experiments are widely used nowadays in order to determine the optimum formulation of various components and blending ratios in multicomponent composite materials. The specific objective is to determine, analyze and optimize the most preferred mixture proportion in a composite product at the lowest price [30,31].

The main aim of this research is to analyze the effect of polyester waste fibers, olive root fibers as reinforcement and coir pith as filler on the mechanical properties (tensile strength, flexural strength and impact strength), weathering effects (water absorption and density loss) and the thermal properties (coefficient of linear thermal expansion and thermogravimetric analysis) of hybrid composites based on a polyester matrix. After modeling the mixture design, the aim is to determine the optimized component proportion for the experimental responses through maximizing and minimizing each component. The mixture design analysis was applied through Minitab statistical software in order to model the relationship between input variables and the experimental responses.

## 2. Materials and Methods

### 2.1. Materials

**Figure 1:**
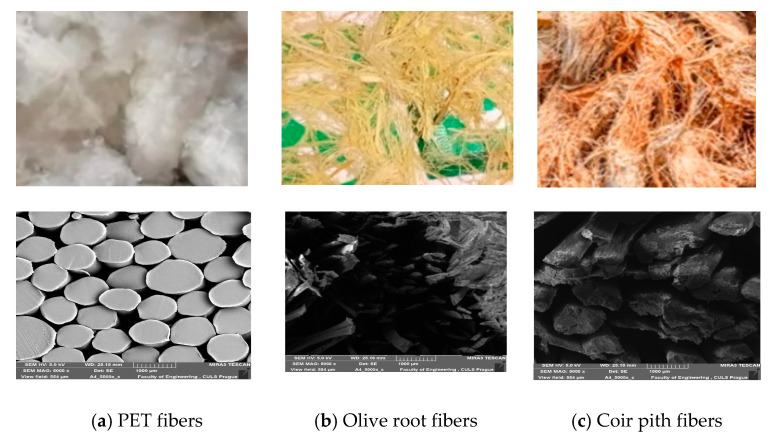
SEM images of fibers used.

Polyester waste fibers of varying length in the range of 2–5 mm was provided by Diamond export industries Pvt. Ltd., Jaranwala Road, Khurrianwala, Faisalabad, Pakistan. This waste was removed in the rinsing process during the manufacturing of fabric. Olive root sticks were procured from a local market in Faisalabad, Pakistan. The olive root fibers were obtained by beating/pressing the sticks and extracting the fibers manually. The fibers were cut into a staple of 2–5 mm. Coconut husk was collected from local vendors and was dried under the sun to remove the moisture from them. The raw materials used are shown in Figure 1.

**Figure 2:**
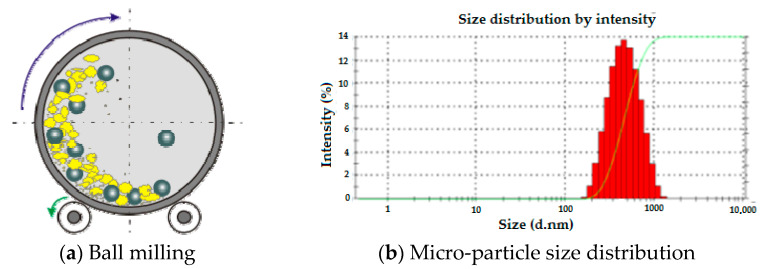
Ball milling to prepare coir pith micro-particles.

The coir pith was extracted manually and cleaned from dust and dirt by washing with distilled water. Fibers were chopped into 2 mm length. Selected samples were treated with 2% NaOH solution for 4 h at room temperature. Further, the alkali-treated samples were thoroughly washed with distilled water, so as to leave no trace of alkali. The alkali treatment removes pectins, fats, lignin and hemicellulose from the fiber, thereby increasing the percentage of cellulose content. This also improves the adhesivity of coir surface with the resin in a composite. In order to obtain the required particle size, alkali-treated coir pith was subjected through the ball mill process as shown in Figure 2. Z-average size of ball-milled coir pith micro-particles was measured to be 312 d.nm as per stander protocol on Malvern-zeta sizer.

The commercially available unsaturated polyester resin was obtained from Changzhou Rixin Resin Co., Ltd., China. Unsaturated polyester resin is a thermosetting polymer, which is chemically similar to epoxy resin and vinyl ester resins. It has excellent and long-term durability to water. It offers adequate resistance to different chemicals, to a large range of substances, from vegetable oil to sulfuric acid. The list of some chemicals’ resistance along their resistive level is shown in Table 1.

Unsaturated polyester resin also exhibits adequate resistance against extreme weathering conditions like rain, ice, snow, strong winds, high temperature, and UV rays. The physical, mechanical and thermal properties of polyester resin are given in Table 2.

COBALT was used as an initiator and methyl ethyl ketone peroxide (MEKP) as a curing agent were procured from local market.

### 2.2. Methods

Twelve samples of hybrid composites were prepared with different compositions of reinforcement materials by weight percentage as given in Table 3 of the experiment design. The mold of size (340.8 mm × 340.8 mm × 3 mm) was used, and mold-releasing wax was applied to it before developing the samples. Polyester waste fibers and olive root fibers were blended in different ratios (0:100, 33:67, 67:33 and 100:0). The reinforcing fibers were loaded as per the required blend ratio and were arranged in the form of a matt inside the mold. The unsaturated polyester resin was mixed with 1 wt% COBALT as an accelerator and 0.5 wt% ethyl methyl ketone peroxide (EMKP) as a matrix curing agent. Coir pith micro-particles were used as filler in the polyester matrix at three different levels (0%, 5%, and 10%) of the overall matrix weight. The matrix with micro-particles was poured and applied by the hand lay-up method. The total aggregate of the loaded mixture components of polyester waste fibers (*Xp*), olive root fibers (*Xo*), and coir pith (*Xc*) is equal to 100 wt% as shown in Table 3. After impregnation, the specimens were subjected to compression molding, under a pressure of 10 kg/cm^2^ at a temperature of 100 °C for 15 min. All samples were stored post-curing at room temperature for 24 h, and then cut into required testing specimens. For all the samples, a thickness of 3 ± 0.1 mm was maintained. The commercial fiber glass sample which was used for comparison purposes also has the same thickness.

### 2.3. Characterization

#### 2.3.1. Mechanical Testing

Tensile, flexural and impact tests of the hybrid composite were carried out according to ASTM D3039, ASTM D7264 and ASTM D 790-02 respectively. The specimen testing was performed under controlled environmental conditions at 23 °C and relative humidity at 65%. The tensile test of the specimens of size (25 mm × 203 mm) was carried out on a universal testing machine (model UMT Z 100 Allroundline, Zwick–Germany). The flexural tests (3-point bending) of specimens with size (13 mm × 120 mm) were carried out using a thickness to span ratio of 24. The impact test was conducted on a pendulum impact tester (model HIT5.5P, ZWICK–Germany).

#### 2.3.2. Environmental Degradation/Weathering Related Properties

The environmental degradation or the impact of weathering on the hybrid composites for outdoor exposure was tested according to ASTM D2565-99. The test was conducted on xenon arc weather-Odometer (Atlas Ci 4000) in two cycles. In the first cycle of 18 h, the specimen was exposed to light for 102 min, and water was sprayed for 18 min. Black panel temperature was maintained at 40 °C, having a radiant flux of 41.5 W·m^−2^ in the range of 300 to 400 nm. In the second cycle, the specimen was exposed in the dark with a relative humidity of 95% with no water spray for 6 h at 38 °C black panel temperature. In order to analyze the impact of weathering, the density of composite samples was measured before and after the weathering condition. Further, the percentage decrease in the total density of composite samples was calculated. Surface images were analyzed in order to visually examine the change in the surface of hybrid composite samples both before and after the weathering conditions. The water absorption behavior of hybrid composites was studied according to ASTM D570. The sample was cut into a circular disk of 5 cm diameter. Firstly, the specimen samples were dried at 40 °C for 24 h in an oven. All the specimens were then cooled at room temperature for 30 min. The conditioned specimens were then immersed in distilled water at 23 °C for 24 h. Before and after immersion, the specimens were weighed to the nearest value of 0.001 g. The water absorption capacity was calculated by weight difference, and using the following equation.

Water absorption capacity,

(1) $$w\%=\left(\frac{w_{t}-w_{0}}{w_{0}}\right)\times 100$$

$w_{0}$ = oven-dry weight, $w_{t}$ = weight of specimen after immersion.

#### 2.3.3. Thermal Properties

The thermal properties (coefficient of linear thermal expansion (CTE) and thermo-gravimetric analysis TGA) of the hybrid composite were determined as per standard procedures. The effect of temperature on thermal expansion of the specimens was studied according to ASTM E831 of thermal expansion on DIL801L Dilatometer–TA Instrument with the temperature ranging from room temperature to 150 °C. Thermogravimetric analysis (TGA) of the samples was carried out according to ASTM E1131 on NETZSCH TG 209F1 Libra. A 5 mg sample size was used to determine the TGA parameters. Thermogravimetric analysis was carried out under an oxygen environment from room temperature to 600 °C with a rate of increase of 10 °C/min. The effect of increasing temperature on the degradation profile was studied and peak degradation temperatures were recorded.

## 3. Results and Discussion

In this research, Mixture Design Analysis was used to forecast corresponding responses, i.e., mechanical properties (tensile strength, flexural strength, impact strength), environmental effects (loss of density after exposure to weathering chamber and water absorption) as well as coefficient of thermal expansion by the input of component proportion, e.g., polyester waste fibers (*Xp*), olive root fibers (*Xo*) and coir pith filler (*Xc*). The effect of polyester waste fibers and olive root fiber as reinforcement and coir pith as incorporating filler into polyester resin on its mechanical, environmental, and thermal properties are evaluated. The responses are the functions of properties of different components in the mixture. The obtained experimental results for mechanical properties, environmental degradation properties, and thermal properties are shown in Table 4. At 95% (α = 0.05) confidence level ANOVA tables were generated to determine the results. According to the *p*-value (probability value), the significance level of each term was determined. If the *p*-value will be more than 95% (α ≤ 0.05) each input component will have a significant effect on response and in case if probability value will be less than 95% (α ≥ 0.05) then the influence could not be considered significant and should be rejected from the final analysis because they do not have a significant effect. Moreover, the null hypothesis will be eliminated.

The experimental results reflect the influence of fiber properties, fiber volume fraction and interfacial strength on the mechanical, thermal and environmental properties of the hybrid composites. The constituents of the hybrid composites are PET fibers, olive root fibers, coir pith micro-particles and polyester resin. The individual component properties and their corresponding proportion play a vital role in deciding the performance of the hybrid composites. A blend proportion of 67:33 for PET:Olive root fibers results in the highest tensile strength in the composite samples. The PET fibers are stronger than olive root fibers and thus as the olive root fiber proportion increases, the tensile strength decreases. The addition of 12.67% coir pith micro-particles, leads to an increase in tensile strength. As the proportion of coir pith micro-particles further increases, there is a decrease in the overall fiber component in order to maintain the total content of reinforcement constant. Based on the Halpin–Tsai model, the tensile strength decreases [32,33]. With respect to the tensile modulus of the hybrid composite samples, they are inversely proportional to the tensile deformation of individual components. The olive root fibers are less extensible as compared to PET fibers. Thus, the increasing proportion of olive root fibers, leads to an increase in the tensile modulus. An increase in coir pith particles in the mixture results in a decrease in fibrous components. Therefore, a decrease in tensile modulus is observed.

The flexural properties are very much similar to tensile properties. The bending deformation in a composite sheet, results in tensile deformation on the outer surface and longitudinal compression on the inner surface. The observed behavior is following the Halpin–Tsai model equations [32,33]. A blend proportion of 58.52% (PET), 28.81% (Olive root fiber) and 12.67% (Coir pith particles) results in the highest tensile and flexural strength.

Impact performance is a multiaxial deformation, unlike tensile or bending. It depends on the mechanical performance of components as well as their interaction in the radial and thickness direction. PET fibers being stronger than other reinforcing components in the mixture, are very much dominant in deciding the impact energy absorption. Further, a highly extensible fiber enables better absorption of the momentum of impact. Thus, the higher proportion of PET, results in higher impact strength. The addition of coir pith micro-particles results in an increase in impact strength, despite the corresponding decrease in the fibrous component. This is quite different from the observations regarding tensile and bending performance. This behavior can be attributed to the interfacial bonds provided by the micro-particles with the resin component and thus improvement in the multiaxial resistance to impact. This behavior is not so prominent under uniaxial deformations, e.g., tensile or bending.

Water absorption is dependent on the hygroscopic component proportion. Both olive root fibers, as well as coir pith, are water-absorbing components, unlike PET fibers. Thus, a decreasing proportion of PET fibers and an increasing proportion of olive root fiber and coir pith particles result in an increase in water absorption capacity. In comparison to the other two components, the coir pith particles are more porous and thus are able to absorb a higher amount of liquid water. An increasing proportion of coir pith micro-particles results in increase in water absorption capacity, The Halpin–Tsai model is validated by these observations [33].

Due to the weathering treatment, there is a degradation of some components which results in loss of weight and an increase in the volume of the composites. The decrease in density is dependent on the proportion of degradable components in the mixture. Since olive root and coir pith are biodegradable components, a higher proportion of such materials leads to more decrease in density.

The coefficient of thermal expansion (CTE) is governed by several models, e.g., the Voigt model [34] and the Reuss model [35]. The overall CTE of hybrid composites is governed by the % fraction, elastic modulus and CTE of individual components. As PET is a thermoplastic fiber, it might deform and melt during heating and thus causing shrinkage of the composite. As the portion of PET fibers is decreased, there is a decrease in the level of thermal deformation. The increase in cellulosic components decreases the level of thermal shrinkage or expansion. Thus, the thermal stability of hybrid composites is improved. The addition of 12.67% coir pith particles reduces the thermal shrinkage in hybrid composites. However, with an increasing proportion of coir particles and a corresponding decrease in fibrous components, there is a significant increase in the thermal expansion coefficient. Unlike fibrous reinforcements, coir pith micro-particles are discontinuous elements. They might not restrict the thermal deformations in the resin phase as efficiently as the fibers. This might be the reason behind higher CTE in coir particle-rich samples. It is consistently observed that decreasing proportion of PET fibers and increasing proportion of olive root fibers results in a decrease in thermal expansion coefficient. Such observations are also supported by reported literature from other researchers [34,36].

### 3.1. Scanning Electron Microscopy (SEM) of Composite Samples

**Figure 3:**
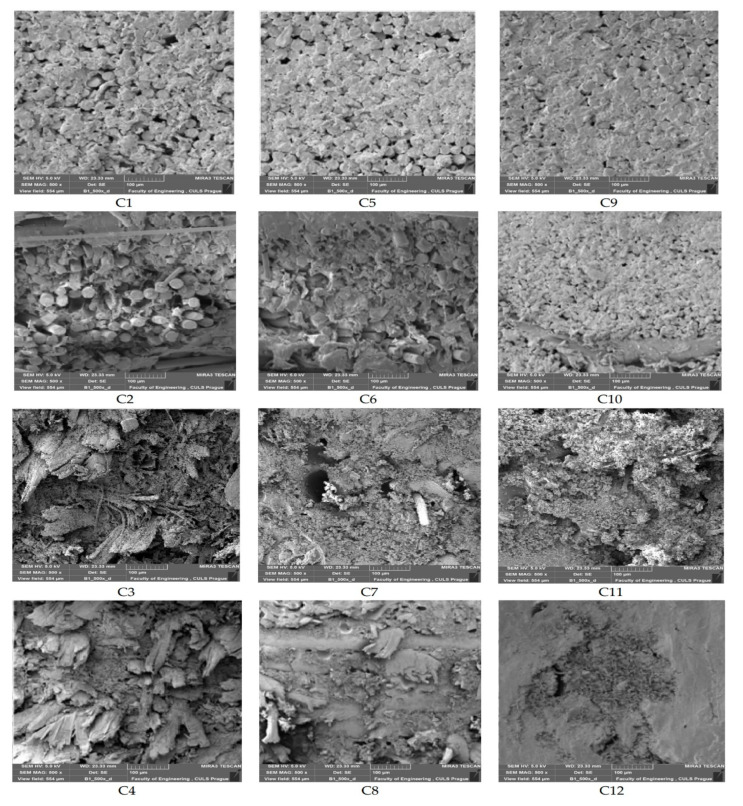
SEM images of tensile tested hybrid composite samples (C1–C12 as in Table 3).

The hybrid composite samples were tested for the mechanical, thermal and environmental/weathering properties. SEM images of tensile-tested hybrid composite samples are shown in Figure 3.

It is visible from the SEM images that the hybrid composites and composites are free from any major defects. The fibrous reinforcement is uniformly impregnated by the matrix phase. When the composite samples contain only one type of fiber, i.e., pure polyester or pure olive root fiber, there is more uniformity. While mixing the polyester and olive root fibers, there are a few minor voids in between the dissimilar fibers. This might be because the diameter, as well as surface morphology of polyester and olive root fibers, is quite different from each other. The pure polyester fiber-based composites show much better impregnation with unsaturated polyester resin. In this case, both the reinforcing fibers as well as resin are based on similar polymers and thus result in better impregnation. On the other hand, olive root fibers are mainly composed of cellulose. Therefore, there are a few minor voids at the fiber–matrix interface. Such voids however are compensated by the hybridization effect in several samples. The addition of coir pith-based cellulosic micro-particles helps in a much better interface with the resin. The uniformly dispersed micro-particles fill all the minor voids around the fibrous phase. Such effect is visible in all the pure as well as hybrid composite samples prepared during this investigation.

### 3.2. Modeling and Statistical Analysis of Experimental Data

Significance models were chosen for all responses using linear, quadratic and special cubic functions of Scheffe’s polynomial equations as given below [37,38].

(2) Y=b1X1+b2X2+b3X3

(3) Y=b1X1+b2X2+b3X3+b12X1X2+b13X1X3+b23X2X3

(4) Y=b1X1+b2X2+b3X3+b12X1X2+b13X1X3+b23X2X3+b123X1X2X3

where *b*1, *b*2, *b*3, *b*12, *b*13, *b*23 and *b*123 are the coefficients of equations that are determined according to the model. *X*1, *X*2, *X*3, *X*1*X*2, *X*1*X*3, *X*2*X*3 and *X*1*X*2*X*3 are the component proportions in the mixture and Y is the dependent variable (responses). However, for all the responses the quadratic model was chosen to be fit for tensile strength, flexural strength, impact energy, water absorption, thermal expansion and the loss of density. Hence, the quadratic models for three-component mixture design take the final form as given below [39]:

(5) η=β1x1+β2x2+β3x3+β12x1x2+β13x1x3+β23x2x3

where,

*η* = Response (mechanical, environmental or thermal)

*βi’s* = Coefficients of equation, *Xi’s* = Component proportion in mixture.

### 3.3. Regression Coefficient and Quadratic Model for All Mechanical, Environmental and Thermal Responses

Statistical summaries of model responses and regression coefficients are determined using Minitab software version 19.1.1. The regression equations corresponding to the best fit models were selected for the estimation of the corresponding mechanical property. Regression coefficients with the probabilities are shown in Table 5, Table 6 and Table 7 for mechanical, environmental and thermal responses respectively. Moreover, quadratic equations were determined from their Regression coefficients as shown below.

(6) η=17.35Xp+19.21Xo−367.4Xc+8.53XpXo+470.4XoXc

(7) η=33.75Xp+36.81Xo−1140Xc+1854XpXc+1919XoXc

(8) η=6.532Xp+3.905Xo−59.8Xc−53.4XpXc−50.2XoXc

(9) η=3.35Xp+7.41Xo+54.3Xc−3.53XpXo−49.21XpXc

(10) η=1.933Xp+11.916Xo+18.8Xc−8.89XpXo

(11) η=−12.48Xp−12.08Xo+970Xc−1123XpXc−1096XoXc

The terms having not significant probability were eliminated from the final equations. Regression Equations (6)–(8) are for tensile strength, flexural strength, and impact strength respectively. Equations (9)–(11) are used for the estimation of water absorption, loss of density due to weathering, and coefficient of thermal expansion respectively.

### 3.4. Analysis of Variance for Mechanical Responses

ANOVA Table 5 shows the analysis of all mechanical responses.

For the tensile strength (MPa) response, the quadratic model is well fitted with a probability *p* > 99%. Two-component interactions, e.g., Xp*Xo, Xp*Xc and Xo*Xc have probability of *p* = 91%, *p* > 99% and *p* > 99% respectively. The two-component interactions of Xp*Xc and Xo*Xc significantly affect the tensile strength of hybrid composites with a probability of *p* > 99%. Still, Xp*Xo component interaction has an insignificant effect with the probability of *p* = 91% (*p* < 95%). The quadratic model also fits the flexural strength (MPa) with a *p* > 98% as shown in two-component proportion interaction. Both Xp*Xc and Xo*Xc affect the response significantly with *p* > 99% respectively. Therefore, both have the highest degree of influence on flexural strength (MPa). However, Xp*Xo component interaction has not been significantly influential due to lower probability (*p* = 91%). Analysis of variance shows that the quadratic model is also well-fitted for the impact strength of the hybrid composites because it indicates a significant probability of *p* = 94%. Two-component proportion interaction of Xp*Xo has an insignificant effect on impact response with the probability of *p* = 62%. However, other two-component interactions Xp*Xc and Xo*Xc have a strong effect on impact response with the probability of *p* > 98% respectively. Therefore, the quadratic model is considered a good fit for the impact strength of hybrid composites.

#### 3.4.1. Normal Probability Plots of Residuals for Mechanical Responses

**Figure 4:**
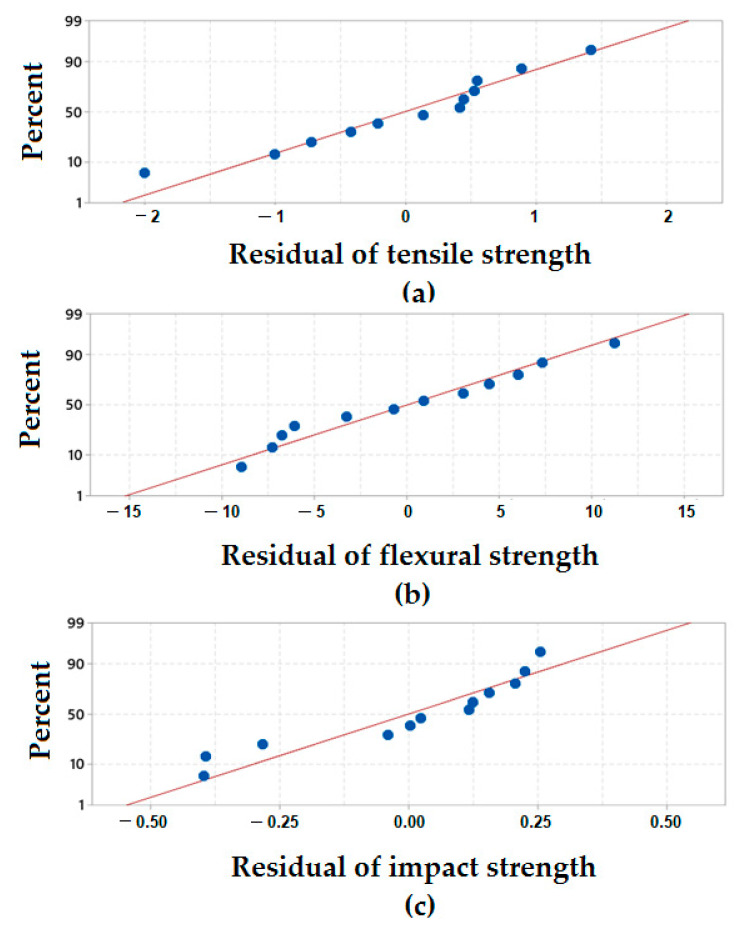
Normal probability plots of residuals (**a**) tensile strength, (**b**) flexural strength and (**c**) impact strength.

Normal probability plots of the residuals determine whether the collected data fit the selected models by the plot point distribution and by comparing the distribution of different samples. If the plotted point distribution is close to the fitted line slope, then the model distribution fits the collected data. For all mechanical properties the residual normal probability plots as shown in Figure 4.

Figure 4a,b show the scatter plots for tensile strength and flexural strength respectively. The plots show that the majority of the points are distributed close to the slope (line) and only a few points are distributed far from the line. In the case of flexural strength, the normal probability plot indicates that approximately all the points are very close to the distribution slope as compared to the plot of tensile strength. Figure 4c shows the normal probability plot of impact strength. It can be noted that the points are somewhat clustered with a relatively lower probability of distribution on the slope line. In general, it can be concluded that all the mechanical responses are quite close to the slope of normal distribution.

#### 3.4.2. Response Trace Plots for Mechanical Properties

**Figure 5:**
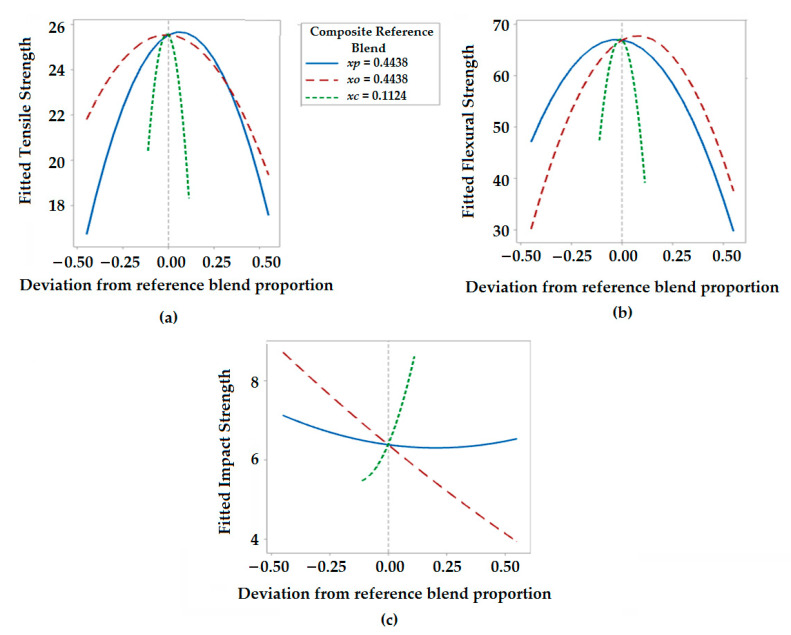
Response trace plots for (**a**) Tensile strength (MPa), (**b**) Flexural strength (MPa), (**c**) Impact strength (KJ/m^2^).

Responses trace plots are also known as component effect plots. These plots help to analyze the effect of each proportion of individual components in a mixture on a selected response. The effect of varying component proportions along the imaginary line connecting the reference mixture on the vertex is shown by response trace curves. For the mechanical properties, response trace plots are shown in Figure 5a–c.

As can be seen in Figure 5a, there is an increase in tensile strength to a magnitude of about 27 MPa with an increasing proportion of polyester waste fibers (*Xp*). The proportion of olive root fibers (*Xo*) is maintained at an optimum level before the tensile strength starts to decrease. Such findings are in accordance with the mechanical properties of polyester and olive root fibers. The polyester fibers are stronger than the olive root fibers and thus dominate their influence on the tensile strength of the composites. The addition of excess fibers beyond a certain limit leads to improper impregnation and a decrease in strength. It is also visible that the proportion of coir pith micro-particles (*Xc*) enhances the tensile strength to an optimum extent and further addition of coir pith decreases the tensile strength. micro-particles provide a very high interfacial area for bonding and thus enhance mechanical properties in a composite system. The behavior is reflecting the findings in literature based on Halpin–Tsai equations [32,33].

Figure 5b shows that flexural strength approaches a maximum magnitude of 78 MPa with an increasing proportion of olive root fibers and a decreasing proportion of polyester fiber in the mixture. The flexural strength is dominated by the olive root fibers as they are coarser and show higher stiffness as compared to polyester waste fibers. Moreover, the flexural strength increases to an optimum level with an increase in the proportion of micro-fillers, and subsequently shows a decreasing trend.

The response trace plot in Figure 5c indicates that the impact strength of hybrid composites linearly decreases with an increased proportion of olive root fibers. Polyester fibers are stronger and have a higher shock-absorbing capacity. Moreover, the coir pith micro-particles have a similar effect on the impact properties of hybrid composites. The micro-fillers improve overall bonding with the matrix and thus enhance the bulk mechanical properties, e.g., absorption of impact stresses. The component ratio is observed to have a synergistic effect on the mechanical properties investigated in this research.

#### 3.4.3. Contour Plots for Mechanical Responses

**Figure 6:**
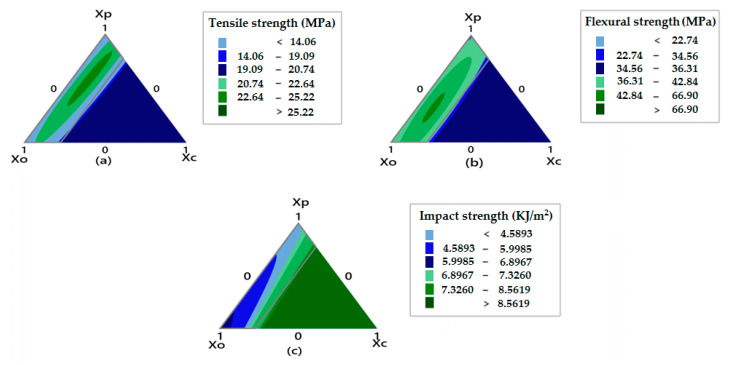
Contour plots for (**a**) Tensile strength, (**b**) Flexural strength, (**c**) Impact strength.

A contour plot is a two-dimensional plot that indicates the effect of varying combinations of mixture components on the magnitude of response. The contour plot shows differently colored regions corresponding to the magnitude of responses. The higher magnitude of response is shown by the highlighted darker-colored regions compared to the lighter-colored regions. The contour plot can calculate the proportions of the components in the mixture and indicate the magnitude of different responses for corresponding color regions. The contour plots for mechanical properties are shown in Figure 6.

The contour plot shown in Figure 6a indicates the highest value for tensile strength (higher than 27.02 MPa) in the darker green region which is at the edge of *Xp* (polyester waste fiber) and *Xo* (olive root fibers). Moving along the contour plot, the highest value for the tensile strength response was 27.02 MPa, as shown in Table 4 for sample C6 (*Xp* = 58.5, *Xo* = 28.81, *Xc* = 12.67). The minimum value for the tensile strength was obtained for the sample C12 (*Xp* = 0.00, *Xo* = 77.72, *Xc* = 22.48) as seen in Table 4. The contour plot of Figure 6b shows the maximum flexural strength which is indicated by the darker green region just like the tensile response plot on the edges of *Xo* (olive root fiber) and *Xp* (polyester waste fiber) with optimum content of *Xc* (coir pith micro-particles). Figure 6c indicates a bigger region of darker green shade which implies that the impact strength is substantially affected by a combination of polyester and olive root fiber proportions. Significant improvement of impact strength can be achieved by increasing the proportion of polyester fibers and coir pith micro-particle content. The results are in agreement with the experimental findings. The trends are similar to theoretical calculation of mechanical properties as reported by other researchers based on Halpin–Tsai models [32,33].

As reported in the literature, remarkable enhancement in mechanical properties was observed in hybrid composites from date palm leaf incorporated recycled poly (ethylene terephthalate) developed by injection molding. Impact strength increased with higher loading of fiber. In addition, tensile strength and flexural strength were enhanced by adding a higher proportion of fiber when compared to the neat matrix. Recycle PET combined with date palm leaf could be a good alternative to obtain eco-friendly products [40]. Hybrid composites fabricated by using wood flour, coir pith powder and corn cob with different ratios in PVC, significantly improved tensile strength, flexural strength and energy absorption properties. The incorporation of coir pith and corn cob reduces voids and cavities in the hybrid composite resulting in the improvement of mechanical properties [41].

### 3.5. Analysis of Variance for Weathering Related Properties

ANOVA Table 6 shows the analysis of variance for water absorption response and loss of density after weathering treatment.

It reveals that the quadratic model is a suitable model for the water absorption behavior of hybrid composites since it predicts the response with a probability value of *p* = 96%. Two-component interactions, e.g., Xp*Xo, Xp*Xc and Xo*Xc have probability of *p* = 96%, *p* = 94% and *p* = 89% respectively. Both Xp*Xo, Xp*Xc affect the water absorption properties of composites in a significant manner. The interaction, Xo*Xc component is insignificant due to its poor probability of *p* = 89%. This means that the water absorption is critically dependent on the proportion of polyester fibers and olive root fibers. Since the proportion of coir pith micro-particles is relatively smaller, it has an insignificant effect on the overall water absorption capacity.

Variance analysis (Table 6), for the percentage decrease in density, indicates that the quadratic model is a significant predictive model with a probability value of *p* > 99%. The two-component interactions of Xp*Xo, Xp*Xc and Xo*Xc have probability value of *p* > 99%, *p* = 40% and *p* = 15% respectively. These observations indicate that the loss of density is mainly governed by the proportions of polyester and olive root fibers in the hybrid composite. The coir pith micro-particles have an insignificant influence on the loss of density due to their lower proportion.

#### 3.5.1. Normal Probability Plots of Residuals for Environmental/Weathering Related Properties

**Figure 7:**
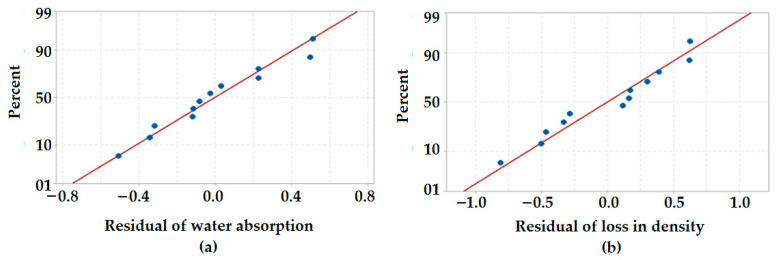
Normal probability plots of residuals for (**a**) water absorption (%), (**b**) density loss percentage.

The normal probability plots of residuals for environmental properties are shown in Figure 7. The normal probability plot in Figure 7a shows that the points are very close to the slope line. This validates the normal distribution of water absorption behavior observed in hybrid composites. Additionally, from Figure 7b, it can be observed that all the fitted points are closer to the distribution slope in case of loss in the density of the composite samples after weathering treatment. This also confirms a normal distribution of the data obtained through the weathering experiment.

#### 3.5.2. Response Trace Plot for Environmental Properties

**Figure 8:**
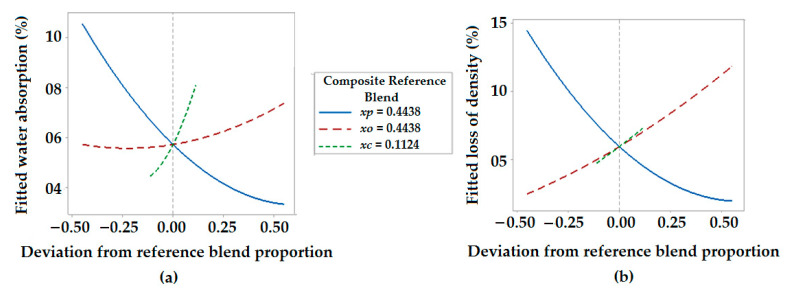
Response trace plot for (**a**) water absorption (%), (**b**) percentage decrease in density.

The response trace plot for the water absorption properties of hybrid composites is shown in Figure 8a. It indicates that with the increase in the proportion of polyester fiber, water absorption decreases, and it increases with an increase in the proportion of both olive root fiber and coir pith micro-particles. Moreover, from Figure 8b, it can be concluded that there is a linear decrease in density after exposure to the outdoor environment based on both the proportion of olive root fibers and coir pith micro-particles. However, the proportion of polyester waste fiber shows a negligible effect on the loss of density of hybrid composites when exposing them to the outdoor environment. Generally, the density of each composite sample decreases but with a different percentage after exposure. As the incorporation of polyester waste fiber decreases and the proportion of olive root fiber and coir pith micro-particles increases, there is a substantial decrement of density.

#### 3.5.3. Contour Plots for Environmental/Weathering Related Properties

**Figure 9:**
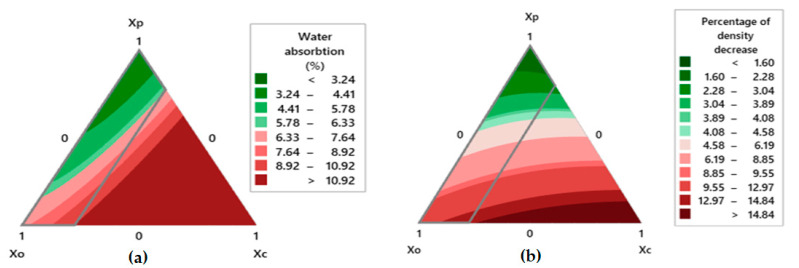
Contour plot for (**a**) water absorption (%), (**b**) percentage loss of density.

Figure 9a shows a contour plot for the water absorption properties of hybrid composites. Moving along the contour plot, it can be observed that the minimum value is indicated in the green region dominated by *Xp* (polyester waste fiber). When moving from this region towards the *Xo* (olive root proportion) and *Xc* (coir pith filler) region, the water absorption increases which is indicated by darker red color. The lowest value as given in Table 4 is about 3.24 (%) for the sample C1 of (*Xp* = 100, *Xo* = 0.00, *Xc* = 0.00). Water absorption behavior increases with the increasing proportion of olive root fiber and coir pith micro-particle filler in the hybrid composite. In practical terms, these natural origin fibers and micro-particles are much more hygroscopic as compared to polyester fibers. The maximum water absorption of hybrid composite was about 10.92% for the sample C12 (*Xp* = 0.00, *Xo* = 77.52, *Xc* = 22.48).

The impact of weathering treatment on the percentage loss of density can be noted in Figure 9b. The maximum decrease in the density can be seen in the darker red region and the edge of *Xo* and *Xc* contour plot. The indication of lower density loss was given in the darker green region at the edge of *Xp*. Maximum value for density loss was about 14.84% for the sample C12 (*Xp* = 0.00, *Xo* = 77.52, *Xc* = 22.48) and the minimum value for the density loss was about 1.60% for sample C1 (*Xp* = 100, *Xo* = 0.00, *Xc* = 0.00). The polyester fiber is least degradable which leads to minimum density loss. Biological origin materials, e.g., olive root fibers or coir pith micro-particles are susceptible to degradation and loss of density. Due to weathering, some of the fibers and micro-particles are degraded and this leads to loss of weight. Further, the volume is found to increase causing a loss of density.

**Figure 10:**
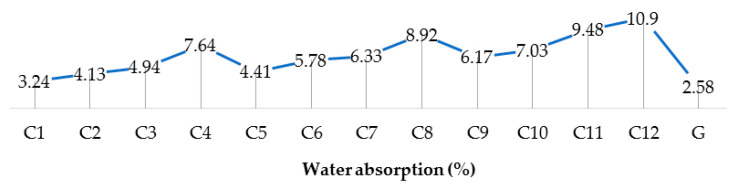
Water absorption (%) of hybrid composites.

**Figure 11:**
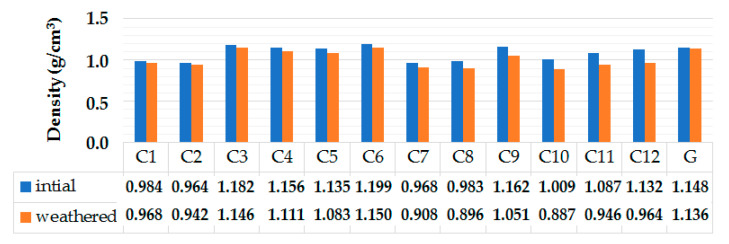
Densities of hybrid composite before and after weathering.

Figure 10 and Figure 11 show the variations of water absorption and change in density before and after weathering for all the hybrid composite samples as compared to fiber glass sunshade panels.

#### 3.5.4. Surface Damage after Weathering Treatment

After weathering treatment, the changes on the surface of each hybrid composite were visually examined. It was observed that the hybrid composite samples having olive root fibers and coir pith micro-particles as major constituents resulted in more surface degradation as compared to composite samples containing polyester waste fibers as a major constituent. Moreover, coir pith micro-particles and olive root fibers protruded on the surface of the composite samples. This is due to the degradation of olive root fibers and coir pith during weathering treatment. Moreover, the observed higher loss in the density of such hybrid composites based on the major proportion of olive root fibers and coir pith micro-particles can be due to the effect of moisture and UV radiation on cellulose, lignin and hemicellulose in these biological origin materials. The hybrid composites rich in cellulosic components degraded due to exposure to a combination of xenon arc radiation and water spray. However, the resistance to environmental degradation of such hybrid composites is significantly improved by increasing the proportion of PET fibers in the composite [23].

### 3.6. Analysis of Variance for Thermal Properties

ANOVA of the thermal expansion properties in hybrid composites is given in Table 7.

The results indicate that the quadratic model is a suitable model to fit the thermal expansion behavior of hybrid composites. It predicts the thermal expansion response with a probability value of *p* = 86%. Two-component interaction Xp*Xo has a probability of *p* = 74% and thus this interaction has a relatively insignificant effect on the thermal response. While two-component interactions of Xp*Xc and Xo*Xc show a significant influence on thermal expansion properties of hybrid composite with a probability value of *p* = 96% and *p* = 94% respectively. Therefore, the quadratic model is a relatively good choice to fit the coefficient of thermal expansion as compared to the linear model. The thermal expansion in a composite system is governed by the Halpin–Tsai models and the hybrid composites in the present investigation follow a similar trend [32,33]. The matrix constitutes the majority component in the composites and thus the thermal properties are dominated by the matrix thermal properties. The addition of micro-particles restricts the thermal deformations in the hybrid composites. Since polyester fiber is thermoplastic in nature, a higher proportion always leads to higher deformation in response to thermal conditions. On the other hand, the presence of a higher proportion of cellulosic fiber like olive root fibers restricts thermal deformation.

#### 3.6.1. Normal Probability Plot of Residuals for Thermal Properties

**Figure 12:**
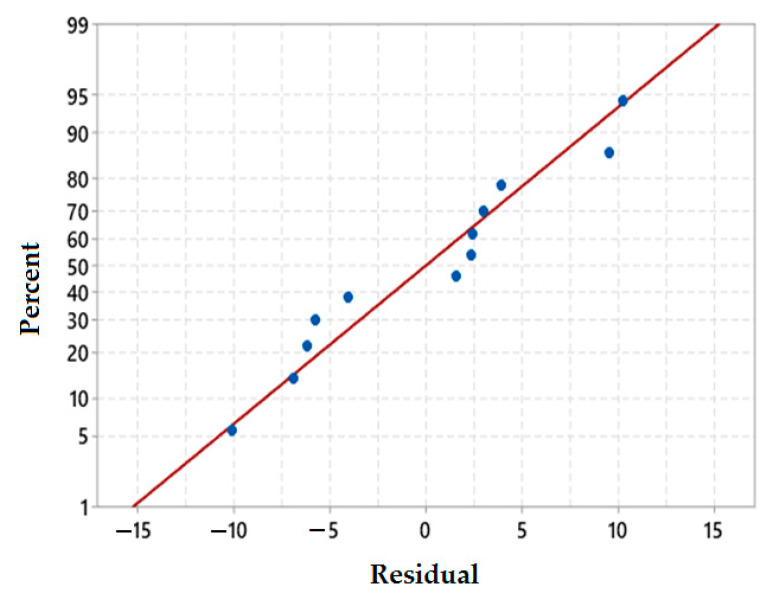
Residual normal probability plot of CTE.

A residual normal probability plot for the coefficient of thermal expansion (CTE), as seen in Figure 12, indicates that five data points out of twelve data points fall very close to the fitted slope line. The remaining points are distributed in groups very close to each other and also close to the probability line. Residual normal probability plot thus validates the distribution of coefficient of a thermal expansion near to normal distribution.

#### 3.6.2. Response Trace Plot for Thermal Properties

**Figure 13:**
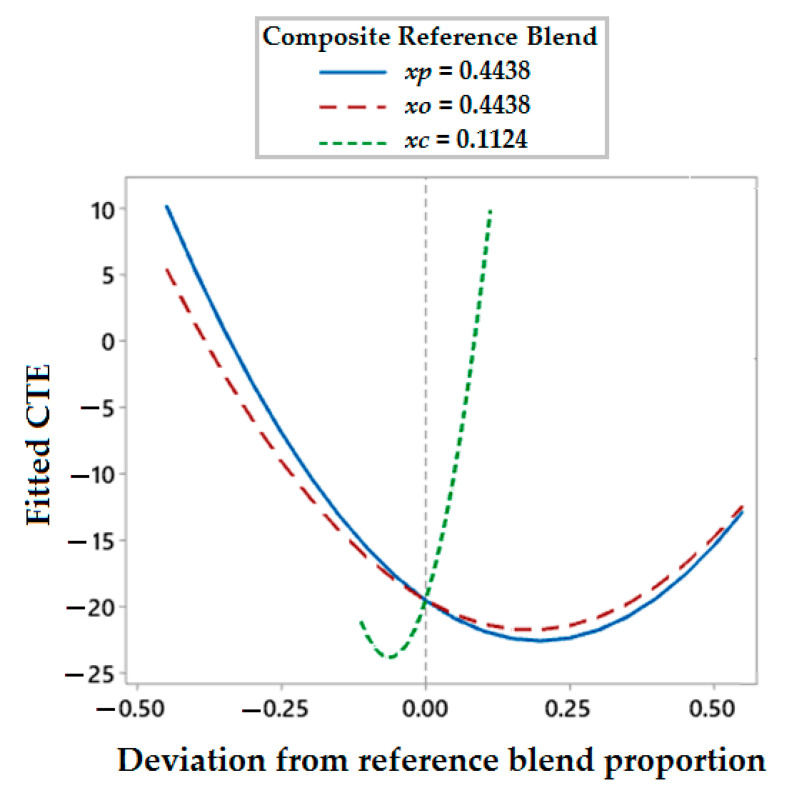
Response trace plot of CTE.

As shown in Figure 13, the response trace plot for the thermal expansion of hybrid composites concludes that the two-component interaction of polyester waste fiber with olive root fiber (Xp*Xo) affects a linear decrement in the thermal expansion behavior of samples to an optimum level. After that the hybrid composites start to expand. Moreover, the addition of coir pith micro-particles enables a linear decrement of thermal expansion coefficient in composites. Both the reinforcing fibers as well as the micro-particle filler provide restrictions to the expansion of the composite by offering resistance at the interfacial region with the matrix. Such behavior is well established by theoretical models using the rule of the mixture as well as modified Halpin–Tsai equations [32,33].

#### 3.6.3. Contour Plot for Thermal Response

**Figure 14:**
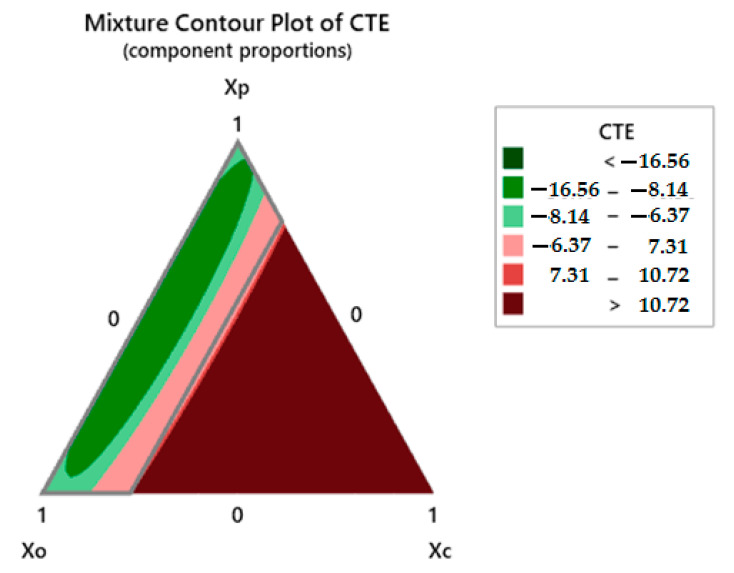
Coefficient of thermal expansion for hybrid composites.

For the thermal expansion behavior, the contour plot is shown in Figure 14.

A higher proportion of *Xp* (polyester waste fiber) offers a negative coefficient of thermal expansion. This tendency of negative thermal expansion was observed for the samples C1 to C8 as shown in Table 3 and Table 4. The minimum negative thermal expansion value was observed for sample C8. These trends indicate that reinforcing fibers actually cause a thermal shrinkage. The shrinkage is reduced as the polyester fiber proportion is reduced. As polyester is a thermoplastic fiber material, it might deform during the increase in temperature and cause this shrinkage. On the other hand, olive root fibers are plant-origin cellulosic materials. They do not tend to deform as the temperature rises. The hybrid composites containing polyester waste fibers, exhibit expansion in linear trend from 25–75 °C. As the temperature further increases, the rate of contraction in the composite increases constantly up to 150 °C and the coefficient of thermal expansion turns negative. However, the hybrid composites containing a major proportion of olive root fibers and coir pith micro-particles in the mixture expand linearly up to 150 °C and show relatively lower coefficients of thermal expansion. These observations indicate that biological origin (cellulosic) reinforcements and fillers provide thermal stability and shape retention to a hybrid composite as opposed to thermoplastic fibers like polyester. Guangfa et.al also observed that polyester fiber starts to contract and the coefficient of thermal expansion turns into a negative value as the temperature increased above 220 °C [42].

### 3.7. Thermogravimetric Analysis of Hybrid Composites and Composites

**Figure 15:**
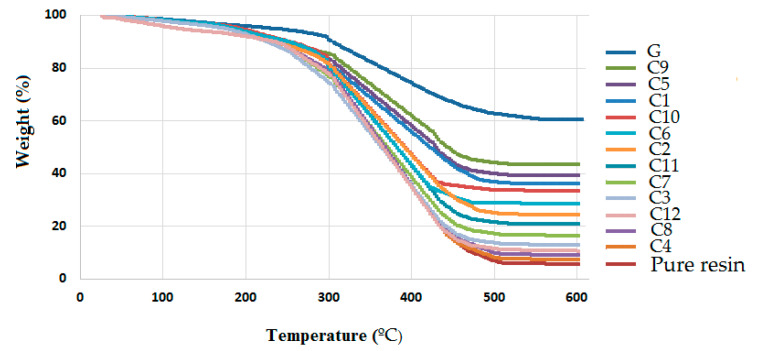
TGA–curves of hybrid composites.

TGA (Thermo-Gravimetric Analysis) results of the hybrid composites describe the thermal stability at elevated temperatures. It can be observed that thermal stability is significantly affected by a mixture of polyester waste fiber, olive root fiber and coir pith micro-particles. The TGA curves shown in Figure 15 explain the weight loss at the onset of thermal degradation.

The TGA results of composite samples as shown in Figure 15 indicate weight loss of 0.3% to 6% of the original weight between 100 °C and 200 °C respectively due to elimination of moisture in the matrix. At about 450 °C, the weight loss was between 15% to 57% due to the degradation and volatilization of some components in the matrix. Subsequently, there is no further loss of weight up to 600 °C. This trend is visible for all the hybrid and non-hybrid composites as well as composites in the present study.

It can be stated that the thermal stability of hybrid composites is significantly influenced by the matrix and different reinforcement materials in the component mixture. The thermal decomposition takes place in a three-step process for each composite sample as can be seen in the TGA curves. The decomposition temperature for the olive root fiber-based composite samples was lower than the pure polyester fiber-based samples. This may be due to higher moisture content and volatile substances in natural origin cellulosic fibers as compared to polyester fibers. In the first stage, a small mass loss was observed for all samples between 210 °C to 310 °C due to the degradation of organic elements in the composites. For the second stage of decomposition, in the temperature range from 310 °C to 370 °C, the hemicellulose component in the olive root fiber may be responsible. The third peak at about 420 °C was mainly due to the degradation of the unsaturated polyester matrix.

Table 8 shows the maximum temperature of decomposition, mass loss (%) and char (%) at 600 °C for all the samples.

The hybrid composites show changes in the process of decomposition due to varying component interactions. The composites with a higher proportion of polyester waste fibers show better thermal stability up to a higher peak temperature of degradation. Hybridization with cellulosic fibers reduces the peak decomposition temperature. On the other hand, the addition of scale fillers in the matrix improves thermal stability. This is mainly because the micro-particles derived from cellulosic fibers, e.g., coir are free from volatile components. These micro-particles are highly crystalline and are free from moisture. The incorporation of scale fillers enables dissipation of heat in the composite system and the degradation is prohibited up to a higher temperature level. When the proportion of micro-fillers was increased from 5% to 10%, a further increase in peak temperature was observed.

The mass loss was observed to be reduced by the hybridization of polyester and olive root fibers. Mass loss in polyester-rich composites was lower as compared to olive root fiber-rich samples. The combination of PET and cellulosic materials in a hybrid composite works in a synergistic manner and they compensate and complement the weaknesses of each other. The use of micro-particles from cellulose origin materials proves to be beneficial in order to improve thermal stability and reduce overall mass loss at elevated temperatures.

The mass loss (%) of the hybrid composites, as well as composites, was higher as compared to commercial fiber glass sunshade panels though the glass panel exhibits a slightly higher peak temperature of degradation.

### 3.8. Optimization for all Responses

The optimal mechanical properties (tensile strength, flexural strength and impact strength), environmental/weathering properties (water absorption and density loss) and the thermal properties (coefficient of thermal expansion) of the hybrid composites were estimated using an optimization approach for each dependent response. The mechanical responses were maximized, environmental properties and thermal expansion properties were minimized. By using this approach, the best combination was found to be *Xp* = 70.83%, *Xo* = 15.16% and *Xc* = 14.01%.

## 4. Conclusions

In this study, an attempt was made to fabricate hybrid composite materials using polyester waste fibers, olive root fibers and coir pith particles as reinforcement in unsaturated polyester resin. Micro-particles obtained from coir pith by ball milling process were used as filler in the polyester resin. The mechanical, thermal, and weathering-related properties were determined for all fabricated hybrid and non-hybrid composite samples. These hybrid composites were then compared with commercial glass fiber-based composite panels used as a sunshade in interior applications.

Mixture design analysis was used to optimize the component proportions in the hybrid composite. For all mechanical responses (tensile strength, flexural strength, and impact strength) environmental properties (water absorption and density loss after weathering) and for the coefficient of thermal expansion, statistical models were developed which show good validity with experimental results. The best formulation was determined at 70.83 wt%, 15.16 wt% and 14.01 wt% of polyester waste fibers, olive root fibers and coir pith fillers, respectively.

Among all the hybrid composites, sample C6 showed the maximum tensile strength and maximum flexural strength. In comparison with commercial glass fiber-based composite, sample C6 reached 30.42% of its tensile strength and almost half of its flexural strength. Maximum tensile strength, flexural strength and impact strength in the developed hybrid composite can be achieved by optimum mixture ratio of polyester waste fiber, olive root fiber and coir pith micro-particles.

The maximum impact strength of the developed hybrid composites was obtained in samples C9 and C10. However, by comparison with commercially available glass fiber composite, C9 composite showed almost 15% of its impact strength. The impact strength increased as the mixture proportion of polyester waste fiber and coir pith micro-particles was increased.

With respect to the environmental/weathering-related properties, C1, C5, and C9 showed the minimum water absorption% as well as density loss which is almost nearer to the commercially available glass fiber composite. Comparing the water absorbency of commercially available glass fiber composite and the developed composite sample (C1), it is found that there is a minute difference in their water absorption capacity. The commercially available glass fiber panel loses 1.2% of its density after weathering while the developed composite C1 lost 1.6% of its density. From the results, it was found that the developed hybrid composite containing a major proportion of polyester waste fiber along with other cellulosic components can be preferred for outdoor applications.

At 150 °C, sample C12 showed the minimum value of thermal expansion. The hybrid composite sample C9 containing a major proportion of polyester waste fiber and coir pith micro-particles in the mixture results in a minimum weight loss (%) and a maximum char (%) at 600 °C as per the TGA results. It resulted in 45% residual char as against the commercial glass fiber composite giving about 62% char. Based on mechanical, thermal and weathering properties, the hybrid composites from polyester waste fiber, olive root fiber and coir pith micro-particles can be categorized separately for outdoor (sunshade) and indoor (interior) applications. These can be used in construction and structural applications at the same time addressing issues of waste management and eco-friendliness.

## Figures and Tables

**Table 1:** Resistance against different chemicals.

Chemicals	Resistance Level
Dilute acid	+ + +
Dilute alkalis	+ + +
Oil and greases	+ +
Aliphatic hydrocarbons	+
Aromatic hydrocarbons	+
Halogenated hydrocarbons	+
Alcohols	+ + +

Resistance levels: + + + + very good, + + + good, + + moderate, + poor.

**Table 2:** Properties of unsaturated polyester resin.

Properties	Value
Physical State	Liquid at 25 °C
Color	Yellowish
Density, g/cm^3^	1.12 at 20 °C
Viscosity, mPoise	300–600 at 25 °C
Storage Temperature	15–25 °C
Barcol Hardness	40
Tensile Strength, MPa	12
Tensile Modulus, GPa	2.1
Elongation at break, %	2.1
Flexural Strength, MPa	25
Flexural Modulus, GPa	0.9
Heat deflection temperature (HDT,1.80 MPa), °C	80
Glass transition temperature (Tg), °C	115–120

**Table 3:** Design of experiment.

Samples	*Xp*	*Xo*	*Xc*
C1	100.00	0.00	0.00
C2	67.00	33.00	0.00
C3	33.00	67.00	0.00
C4	0.00	100.00	0.00
C5	87.33	0.00	12.67
C6	58.52	28.81	12.67
C7	28.81	58.52	12.67
C8	0.00	87.33	12.67
C9	77.52	0.00	22.48
C10	51.94	25.58	22.48
C11	25.58	51.94	22.48
C12	0.00	77.52	22.48

**Table 4:** Properties of samples investigated.

Sample Code	Tensile Strength (MPa)	Tensile Moduli (GPa)	Flexural Strength (MPa)	Flexural Moduli (GPa)	Impact Strength (KJ/m^2^)	Water Absorbtion (%)	Initial Density (g/cm^3^)	Weathered Density (g/cm^3^)	CTE (10^−6^/K)
C1	16.93	3.25	36.31	1.14	6.6886	3.24	0.984	0.968	−16.56
C2	20.74	3.71	48.32	1.61	5.9985	4.13	0.964	0.942	−11.86
C3	16.58	3.97	35.83	1.84	4.5893	4.94	1.182	1.146	−9.1
C4	15.46	5.21	34.84	2.02	4.0293	7.64	1.156	1.111	−8.14
C5	25.22	2.85	79.02	2.12	7.0858	4.41	1.199	1.15	−9.86
C6	27.02	3.29	86.90	4.11	6.8967	5.78	1.135	1.083	−8.25
C7	24.64	3.80	73.42	4.46	6.2576	6.33	0.968	0.908	−6.37
C8	22.32	3.86	55.22	5.44	5.6868	8.92	0.983	0.896	−4.72
C9	17.09	2.31	34.74	1.25	9.1548	6.17	1.162	1.051	11.64
C10	19.53	2.62	44.56	1.93	9.0445	7.03	1.009	0.887	10.72
C11	16.76	2.81	33.22	3.35	8.5619	9.48	1.087	0.946	8.76
C12	14.86	3.23	31.90	3.87	7.3260	10.9	1.132	0.964	7.31
G	88.83	10.66	158.66	4.99	64.61	2.58	1.148	1.136	3.4

G-Fiber glass commercial sample.

**Table 5:** Analysis of Variance for Tensile strength (MPa), Flexural strength (MPa), and Impact Strength (KJ/m^2^).

Source	DF	Seq SS	Adj SS	Adj MS	F-Value	*p*-Value
**Tensile strength Regression**	5	130.971	130.971	26.194	16.48	0.002
Linear	2	8.081	101.697	50.848	32.00	0.002
Quadratic	3	122.889	122.889	40.963	25.78	0.001
Xp*Xo	1	6.537	6.913	6.913	4.35	0.082
Xp*Xc	1	26.506	105.793	105.793	66.57	0.000
Xo*Xc	1	89.846	89.846	89.846	56.54	0.000
Residual Error	6	9.535	9.535	1.589		
Total	11	140.506				
**Flexural strength Regression**	5	2099.31	2099.3	419.86	5.31	0.033
Linear	2	282.76	1490.1	745.05	9.43	0.024
Quadratic	3	1816.55	1816.5	605.52	7.66	0.018
Xp*Xo	1	312.47	322.4	322.42	4.08	0.090
Xp*Xc	1	9.61	1395.8	1395.85	17.66	0.006
Xo*Xc	1	1494.47	1494.5	1494.47	18.91	0.005
Residual Error	6	474.13	474.1	79.02		
Total	11	2573.44				
**Impact strength Regression**	5	28.1916	28.1916	5.63833	55.56	0.000
Linear	2	26.8809	6.2415	3.12073	30.75	0.081
Quadratic	3	1.3107	1.3107	0.43692	4.31	0.061
Xp*Xo	1	0.0949	0.0902	0.09024	0.89	0.382
Xp*Xc	1	0.1915	1.1597	1.15968	11.43	0.015
Xo*Xc	1	1.0243	1.0243	1.02428	10.09	0.019
Residual Error	6	0.6089	0.6089	0.10148		
Total	11	28.8005				

**Table 6:** Analysis of Variance (component proportions) for water absorption (%) and decrease in density (%).

	Source	DF	Seq SS	Adj SS	Adj MS	F-Value	*p*-Value
**Water absorption (%)**	**Regression**	5	58.1772	58.1772	11.6354	62.11	0.000
	Linear	2	55.2076	11.7230	5.8615	31.29	0.081
	Quadratic	3	2.9696	2.9696	0.9899	5.28	0.040
	Xp*Xo	1	1.2039	1.2183	1.2183	6.50	0.043
	Xp*Xc	1	1.1209	0.9798	0.9798	5.23	0.062
	Xo*Xc	1	0.6448	0.6448	0.6448	3.44	0.113
	Residual Error	6	1.1241	1.1241	0.1873		
	Total	11	59.3013				
**Loss in density (%)**	**Regression**	5	222.356	222.356	44.4711	110.49	0.000
	Linear	2	208.552	61.435	30.7177	76.32	0.014
	Quadratic	3	13.803	13.803	4.6012	11.43	0.007
	Xp*Xo	1	7.493	7.497	7.4972	18.63	0.005
	Xp*Xc	1	6.295	0.126	0.1259	0.31	0.596
	Xo*Xc	1	0.016	0.016	0.0157	0.04	0.850
	Residual Error	6	2.415	2.415	0.4025		
	Total	11	224.770				

**Table 7:** Analysis of variance for the coefficient of thermal expansion.

	Source	DF	Seq SS	Adj SS	Adj MS	F-Value	*p*-Value
**Thermal expansion**	**Regression**	5	2199.86	2199.9	439.97	5.57	0.030
	Linear	2	1573.42	645.9	322.96	4.09	0.176
	Quadratic	3	626.43	626.4	208.81	2.64	0.144
	Xp*Xo	1	113.86	117.4	117.39	1.49	0.269
	Xp*Xc	1	24.87	512.1	512.10	6.48	0.044
	Xo*Xc	1	487.71	487.7	487.71	6.17	0.048
	Residual Error	6	474.23	474.2	79.04		
	Total	11	2674.08				

**Table 8:** TGA results for hybrid composites.

Samples	T MAX (°C)	Mass Loss (%)	Char (%) at 600 °C
C1	454	62	38
C2	438	75	25
C3	433	82	18
C4	432	90	10
C5	456	58	42
C6	440	69	31
C7	436	80	20
C8	433	86	14
C9	457	55	45
C10	448	65	35
C11	438	78	22
C12	436	84	16
G	467	38	62

## Data Availability

Not applicable.
